# Endoscopic Transcanal Myringoplasty: A Comparative Analysis of Graft Uptake and Hearing Outcomes Using Butterfly Cartilage, Temporalis Fascia, and a Novel Composite Cartilage Graft

**DOI:** 10.7759/cureus.83597

**Published:** 2025-05-06

**Authors:** Alok Kumar, Deepali Singh, Binayak Baruah, Deb Sanjay Nag, Amrita Jana

**Affiliations:** 1 Department of Otorhinolaryngology, Tata Main Hospital, Jamshedpur, IND; 2 Department of Otolaryngology - Head and Neck Surgery, Manipal Tata Medical College, Jamshedpur, IND; 3 Department of Anesthesiology, Tata Main Hospital, Jamshedpur, IND

**Keywords:** audiometry, cartilage, endoscopy, fascia, myringoplasty, tympanic membrane perforation

## Abstract

Background: Tympanic membrane perforation repair continues to evolve, with a shift toward endoscopic approaches and diverse grafting materials. Subtotal perforations with minimal anterior margins pose a significant clinical challenge. This study evaluated the efficacy of a novel composite graft (partial-thickness tragal cartilage overlaid with temporalis fascia) compared to established techniques: full-thickness tragal cartilage (butterfly graft) and temporalis fascia.

Methods: This observational retrospective study was conducted at a tertiary care setting. The medical records of 70 consecutive patients who underwent endoscopic transcanal myringoplasty were reviewed. Patients were categorized into three groups based on the myringoplasty technique: butterfly cartilage graft, temporalis fascia graft, and composite cartilage graft. Graft uptake rates and changes in air-bone gap (ABG) were compared pre- and postoperatively (at three months).

Results: All three techniques demonstrated high graft uptake rates and statistically significant improvements in ABG. The butterfly graft had a 100% uptake rate, the temporalis fascia graft had an 89.47% uptake rate, and the composite cartilage graft had an 84.62% uptake rate. Mean ABG improvement at three months postoperatively was 10.06 dB for the butterfly group, 9.53 dB for the temporalis fascia group, and 10.23 dB for the composite cartilage graft group. Significant differences were observed between all groups (p < 0.001). The composite graft demonstrated promising results, particularly for subtotal perforations.

Conclusions: Endoscopic transcanal myringoplasty with various graft materials is an effective method for tympanic membrane repair. The butterfly cartilage graft is suitable for small- to medium-sized perforations, and temporalis fascia remains suitable for large- and medium-sized perforations with adequate anterior margins. For subtotal perforations with minimal anterior margins, the novel composite cartilage graft offers a promising alternative with a high potential for successful outcomes. This warrants further investigation in larger prospective studies.

## Introduction

Myringoplasty, the surgical repair of tympanic membrane perforations, is a cornerstone of otologic surgery. Successful outcomes depend on meticulous surgical techniques, appropriate graft selection, and patient-specific factors such as the presence of middle ear infection, Eustachian tube dysfunction, and the overall health status of the patient [1-5]. The choice of grafting material remains a key factor influencing both graft uptake and postoperative hearing outcomes. Historically, the temporalis fascia was the gold standard [6-8], but its use has been challenged by concerns regarding resorption, inconsistent thickness, and the difficulty of obtaining grafts of appropriate size [9,10].

Autologous cartilage grafts, particularly from the tragus, have emerged as a superior alternative [11-13]. Their advantages include superior biocompatibility, resistance to resorption, and better long-term structural integrity [14-16]. Full-thickness cartilage grafts have demonstrated excellent uptake rates but may result in less favorable hearing outcomes due to excessive thickness [17,18]. Partial-thickness cartilage grafts, designed to minimize hearing impact while maintaining good structural support, have also shown promise [19,20]. The "butterfly" graft, a modification utilizing a full-thickness tragal cartilage with carefully scored margins for improved fit, has been shown to provide excellent results [21].

Managing subtotal perforations with minimal anterior margin remnant remains a significant clinical challenge. These perforations are often associated with higher rates of graft failure and poor hearing outcomes when using temporalis fascia grafts alone [22,23]. Therefore, there is ongoing research aimed at identifying optimal graft techniques and materials to address this specific clinical challenge.

This study introduces a novel composite cartilage graft consisting of partial-thickness tragal cartilage (devoid of perichondrium) overlaid with temporalis fascia. This combination aims to leverage the biocompatibility and structural strength of cartilage with the pliable nature and ease of handling of temporalis fascia. Our objective was to compare the efficacy of this novel composite graft with established techniques (butterfly cartilage and temporalis fascia grafts) in terms of graft uptake rates and hearing outcomes in patients with various sizes and types of tympanic membrane perforations.

## Materials and methods

Study design and patient population

This was a retrospective observational study conducted over a two-year period (from September 2022 to October 2024) at the Department of ENT, Tata Main Hospital, Jamshedpur, India. We reviewed the medical records of 70 consecutive patients who underwent endoscopic transcanal myringoplasty for chronic suppurative otitis media (CSOM). Inclusion criteria included patients with dry middle ears for at least one month, central perforations of varying sizes (small, medium, large, and subtotal), and intact mobile ossicular chains. As used by Kaya et al. [10], this is one of the commonest modalities for classification of perforations. Exclusion criteria included patients with squamous-type CSOM, active ear discharge, disrupted ossicular chains, or sensorineural hearing loss. Being a retrospective observational study, as per institutional policy, Institutional Ethics Committee approval or clinical trial registration was not needed.

Surgical techniques

The following three different myringoplasty techniques were employed.

Butterfly cartilage graft: Full-thickness tragal cartilage was harvested and shaped to match the perforation size. The perichondrium was left intact. Grooves were made at the edges to facilitate a snug fit.

Temporalis fascia graft: A temporalis fascia graft was harvested via a postauricular incision. The graft was positioned over the perforation.

Composite cartilage graft: A partial-thickness tragal cartilage graft was prepared using a cartilage slicer, removing the perichondrium from both sides. This thinned cartilage was then overlaid with a temporalis fascia graft. This technique was primarily used for subtotal perforations.

Assessment of outcomes

Pre- and postoperative pure tone audiograms were obtained to assess hearing levels. The primary outcome measure was the change in air-bone gap (ABG) at three months postoperatively. Secondary outcomes included graft uptake rate (successful graft integration without complications) at these same time points.

Statistical analysis

Data were analyzed using the jamovi project (2025), jamovi (version 2.6), retrieved from https://www.jamovi.org. Descriptive statistics were used to summarize the data. Differences in ABG change and graft uptake rates among the three groups were compared using analysis of variance and chi-square tests, as appropriate. A p value of <0.05 was considered statistically significant.

## Results

Patient demographics

Seventy patients were included (38 men, 32 women; mean age, 43 years; range, 20-60 years). Left ear involvement was slightly more common (52%). The distribution of perforation sizes was as follows: small (15%), medium (30%), large (25%), and subtotal (30%).

Graft uptake and hearing outcomes

**Figure 1:**
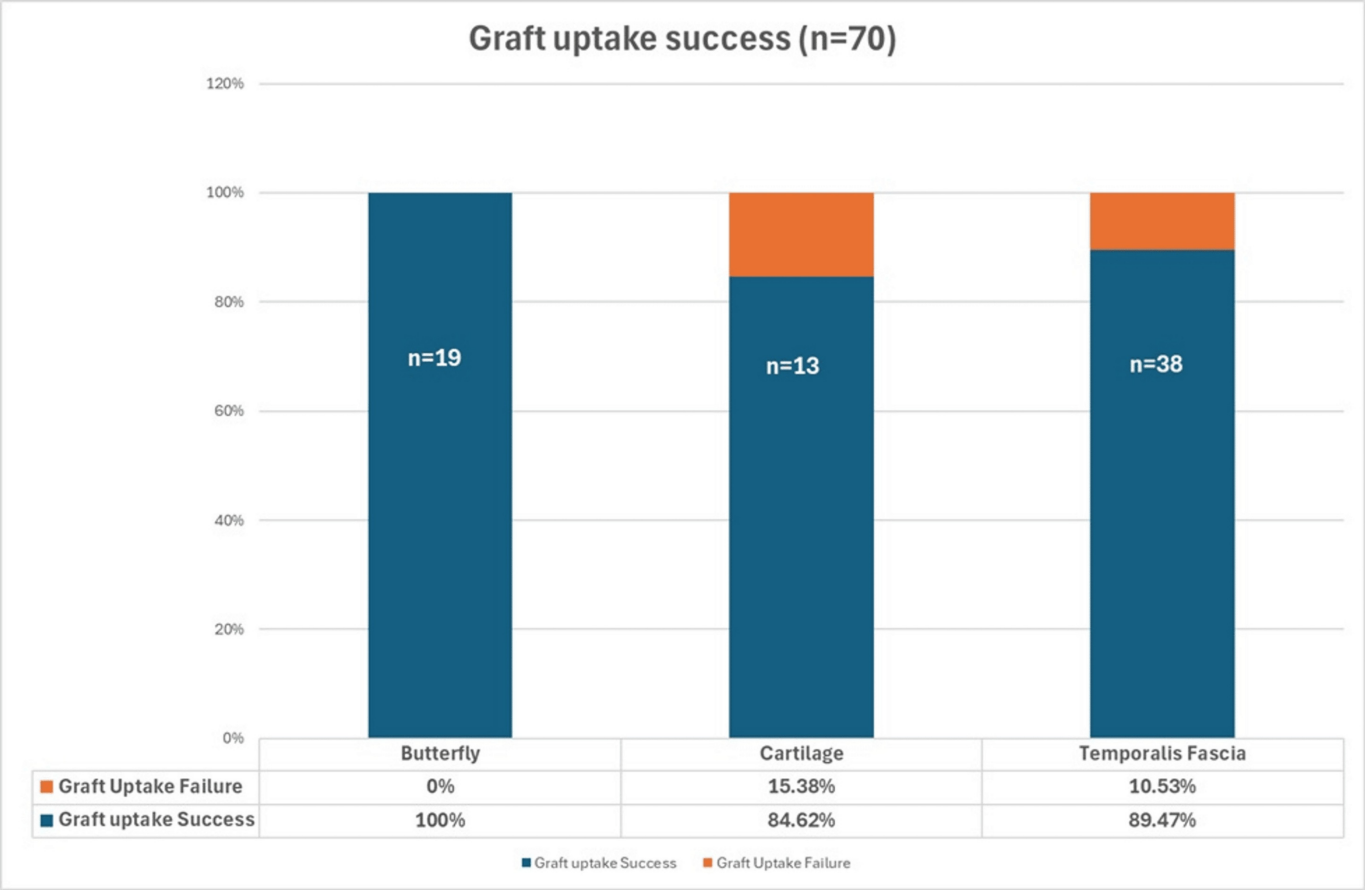
The graft uptake Pearson's chi-square test for proportions shows that the p value is 0.0004895, which indicates that there is a statistically significant difference in uptake percentages among the three groups

The graft uptake rates are depicted in Figure 1. The butterfly graft had a 100% uptake rate; the temporalis fascia graft had an 89.47% uptake rate, and the composite cartilage graft had an 84.62% uptake rate. All differences between the groups were statistically significant (p < 0.001). Table 1 displays hearing outcomes at three months postoperatively. The mean ABG improvement at three months postoperatively was 10.06 dB for the butterfly group, 9.53 dB for the temporalis fascia group, and 10.23 dB for the composite cartilage graft group. The differences among groups were statistically significant (p < 0.001).

**Table 1:** Hearing outcomes at three months postoperatively The p value from the Fisher's exact test is 0.0004998, indicating a statistically significant difference in uptake rates among the three groups. Pairwise comparisons with Bonferroni correction were also performed to identify which groups differ from each other: butterfly vs. cartilage: p value = 0.0001 (significant); butterfly vs. temporalis fascia: p value = 0.0022 (significant); and cartilage vs. temporalis fascia: p value = 1 (not significant) This means that the butterfly group is significantly different from both cartilage and temporalis fascia, while cartilage and temporalis fascia are not significantly different from each other ^*^Statistically significant ABG: air-bone gap; SD: standard deviation

Type of myringoplasty	No. of cases	Preoperative ABG (dB), mean ± SD	Postoperative ABG (dB), mean ± SD	p value
Butterfly	19	41.95 ± 4.49	31.89 ± 6.54	<0.0001^*^
Cartilage	13	56.69 ± 6.58	46.46 ± 8.77	<0.0001^*^
Temporalis fascia	38	51.16 ± 8.04	41.63 ± 9.50	<0.0001^*^
Total	70	49.69 ± 8.63	39.89 ± 9.53	<0.0001^*^

## Discussion

This study provides a detailed comparison of three different myringoplasty techniques. All three techniques demonstrated high graft uptake rates and significant improvements in postoperative ABG, confirming the efficacy of endoscopic transcanal myringoplasty for tympanic membrane perforation repair. The composite graft, though demonstrating a slightly lower uptake rate compared to the butterfly and temporalis fascia methods, provided comparable hearing outcomes, especially for subtotal perforations, which remain challenging for traditional techniques. The retrospective observational study adheres to the Strengthening the Reporting of Observational studies in Epidemiology guidelines.

The high uptake rate (100%) of the butterfly graft is consistent with previous reports in the literature [21]. However, its application is limited to smaller perforations. The temporalis fascia graft, while showing acceptable outcomes, demonstrated statistically lower uptake rates for subtotal perforations, aligning with previous findings [22,23].

The novel composite cartilage graft presented a distinct advantage in managing subtotal perforations with minimal anterior margins. The combination of partial-thickness cartilage and temporalis fascia offers a structurally robust graft that minimizes stiffness for better integration while addressing some of the challenges associated with using temporalis fascia alone.

The observed hearing improvements across all three techniques, albeit with significant differences, are consistent with established surgical outcomes reported in similar studies [24-26]. Further, our results indicate that the composite graft has the potential to superiorly manage subtotal perforations, improving outcomes and potentially reducing surgical revision rates [27-29].

Endoscopic transcanal myringoplasty using various graft materials has shown promising results in treating tympanic membrane perforations. Studies comparing butterfly cartilage and temporalis fascia grafts have demonstrated similar graft uptake rates and hearing improvements [30,31]. Endoscopic and microscopic approaches for butterfly cartilage myringoplasty yielded comparable outcomes, with endoscopy offering advantages such as reduced pain and shorter operative time [32]. For high-risk perforations, cartilage-perichondrial composite grafts performed similarly to temporalis fascia regarding graft uptake and hearing improvement [33]. High-risk perforations are characterized by more than 50% perforation of the tympanic membrane, cases requiring revision, absence or erosion of the malleus handle, edematous or unhealthy middle ear mucosa, and cases with marginal involvement [33]. Across studies, graft uptake rates ranged from 86.2% to 96%, with significant improvements in ABG closure observed postoperatively [30-33]. These findings suggest that various graft materials can be effectively used in endoscopic transcanal myringoplasty, with the choice depending on specific patient factors and surgical preferences.

Limitations

This study is limited by its retrospective nature, the relatively small sample size, and the single-center design. A larger, prospective, multicenter study would be needed to validate our findings and confirm the long-term efficacy of the composite graft.

## Conclusions

Endoscopic transcanal myringoplasty offers a minimally invasive and effective method for repairing tympanic membrane perforations. Our results suggest that the butterfly cartilage graft may be the optimal technique for small- to medium-sized perforations, while the temporalis fascia graft remains suitable for managing large- and medium-sized perforations with adequate anterior margins. However, for subtotal perforations with minimal anterior margins, the novel composite cartilage graft provides a promising alternative with a high potential for successful outcomes. This warrants further investigation in larger prospective studies.
